# Factors responsible for poor sleep quality in patients with chronic obstructive pulmonary disease

**DOI:** 10.1186/s12890-016-0281-6

**Published:** 2016-08-08

**Authors:** Chih-Hao Chang, Li-Pang Chuang, Shih-Wei Lin, Chung-Shu Lee, Ying-Huang Tsai, Yu-Feng Wei, Shih-Lung Cheng, Jeng-Yuan Hsu, Ping-Hung Kuo, Chong-Jen Yu, Ning-Hung Chen

**Affiliations:** 1 Department of Pulmonary and Critical Care Medicine, Linkou Chang-Gung Memorial Hospital, Chang-Gung medical foundation, Taoyuan, Taiwan; 2 Chang-Gung University College of Medicine, Taoyuan, Taiwan; 3 Department of Pulmonary and Critical Care Medicine, Chiayi Chang-Gung Memorial Hospital, Chang-Gung medical foundation, Chiayi, Taiwan; 4 Department of Internal Medicine, E-Da Hospital/I-Shou University, Kaohsiung, Taiwan; 5 Department of Internal Medicine, Division of Thoracic Medicine, Far Eastern Memorial Hospital, New Taipei City, Taiwan; 6 Department of Chemical Engineering and Materials Science, Yuan-Ze University, Taoyuan City, Taiwan; 7 Division of Chest Medicine, Taichung Veterans General Hospital, Taichung, Taiwan; 8 Department of Internal Medicine, National Taiwan University Hospital, Taipei, Taiwan

**Keywords:** COPD, Pittsburgh Sleep Quality Index, COPD Assessment Test, Sleep quality

## Abstract

**Background:**

Sleep disturbance is a common complaint in patients with chronic obstructive lung disease (COPD). However, the factors resulting in sleep disturbance remain unclear. This retrospective, observational, multicenter study aimed to identify the factors associated with sleep disturbance in patients with COPD.

**Methods:**

The study was a retrospective, observational, and multicenter research. Data including age, sex, body mass index, smoking status, COPD inhaler prescribed, clinical symptoms, pulmonary function tests, medical history of comorbidities, and questionnaires were collected. Parameters including demographics, symptoms, medication, severity, functional classification, and comorbidities were correlated with sleep quality scores.

**Results:**

Among 377 patients with COPD, 200 (53 %) patients experienced poor sleep quality (Pittsburg Sleep Quality Index scores > 5). A significant difference in sleep quality, as measured by PSQI scores, was noted between groups based on the 2011 Global Initiatives for Chronic Obstructive Lung Disease (GOLD) classification system. The most common sleep disturbances included “getting up to use the bathroom” (70.3 %), “wake up at night or early morning” (40.3 %), and “cough and snore loudly at night” (15.9 %). The use of inhaled corticosteroids, the presence of wheezing, COPD Assessment Test (CAT) scores, and Modified Medical Research Council (mMRC) dyspnea scale scores positively correlated with poor sleep quality (odds ratio: 1.51, 1.66, 1.09, and 1.30, respectively). Upon multivariate analysis, the CAT score was an independent factor for poor sleep quality in these patients. With the exception of sleep problem items, based on the CAT questionnaire, phlegm was significantly higher in COPD patients with poor sleep quality.

**Conclusions:**

Poor sleep quality is common among patients with COPD and symptoms including wheeze, phlegm, and inhaled corticosteroid use may contribute to poor sleep quality. The CAT score is a good indicator of poor sleep quality in patients with COPD.

## Background

Sleep disturbance is a common symptom in patients with COPD [1, 2] and there are significant differences between the sleep disorders in patients with COPD and those encountered in the general population [3]. Less rapid eye movement (REM) sleep and arousals during periods of desaturation are often noted in patients with COPD [4]. Patients with COPD also become more hypoxemic during sleep than when awake and the hypoxemia during sleep is even greater than that encountered during exercise [5]. Hypoventilation is the major cause of hypoxemia during REM sleep in patients with COPD and there may be additional contributions from ventilation-perfusion mismatches and reductions in functional residual capacity [6].

Recently, Nunes et al. [1] reported that 70 % of patients with COPD had poor sleep quality, and the quality of sleep was the major determinant of quality of life in those patients. In the study by Scharf et al. [2] sleep quality was also associated with quality of life and sleep disturbance predicted poor survival in patients with COPD [7]. However, the factors resulting in sleep disturbance are not well understood.

In patients with moderate to severe COPD, nocturnal desaturation did not correlate with sleep quality or quality of life [8]. In another study, disturbed sleep was correlated with cough and dyspnea symptoms, but not with forced expiratory volume in the first second (FEV_1_) in patients with COPD [7]. In addition, whether current pulmonary medications for COPD, including anticholinergics, theophylline, steroids, and beta2 agonists could improve or impair sleep quality is not well known [9–14]. This study was conducted, therefore, to identify the factors resulting in sleep disturbance in patients with COPD in Taiwan.

## Methods

### Patients

This study was conducted in accordance with the amended Declaration of Helsinki. The study was approved by the Institutional Review Boards of all the hospitals involved.

A retrospective, observational, multicenter research study was performed across six participating hospitals, including four medical centers and two regional hospitals, three in northern Taiwan, one in center-western Taiwan, two in southwestern Taiwan. Between December 2011 and November 2013, patients over 40 years of age with COPD, who were followed in an outpatient pulmonary clinic, were enrolled. Patients with a COPD diagnosis, based on the Global Initiatives for Chronic Obstructive Lung Disease (GOLD) guidelines, were included [15]. The GOLD guidelines are used worldwide in the diagnosis and management of COPD. COPD severity is staging by lung function only according to old GOLD guideline [16]. According to the 2007 GOLD definition: Stage 1: FEV1 ≥ 80 % predicted; Stage: 2 FEV1 < 80 % predicted and ≥ 50 % predicted; Stage 3: FEV1 < 50 % predicted and ≥30 % predicted; and Stage 4: FEV1 < 30 % predicted. In the GOLD guideline updated in 2011, COPD patients are classified into four groups by their symptoms and lung function [15]. COPD symptoms were evaluated by mMRC or CAT as follows: less symptoms patient (A or C) : mMRC 0–1 or CAT < 10; More symptoms patients (B or D): mMRC ≥ 2 or CAT ≥ 10. Low risk patients (A or B): FEV1% predicted ≥ 50 % or COPD exacerbation history 0–1; High risk patients (C or D): FEV1% predicted < 50 % or COPD exacerbation history ≥ 2. The patients with COPD are categorized into A, B, C, and D, four groups.

All participants had pulmonary function tests within one year and at least one COPD Assessment Test (CAT) [17], one Modified Medical Research Council (mMRC) dyspnea scale [18], and one Pittsburgh Sleep Quality Index (PSQI) [19]. Patients with a history of bronchial asthma or malignancy were excluded.

### Data collection

Medical records were retrospectively reviewed and analyzed for the following data: age, sex, body mass index (BMI), smoking status, COPD medications prescribed at outpatient clinics, clinical symptoms, presence of wheezing recorded in the medical charts, pulmonary function tests, and medical history of comorbidities. The COPD maintenance inhaler was defined as an inhaler continuously prescribed for 3 months.

### Measures

The PSQI is a self-rating screening tool that measures quality of sleep. In addition to a total score, PSQI provides seven components of sleep including quality, latency, duration, efficiency, sleep disturbances, use of sleeping medication, and daytime dysfunction. Each component is scored from 0 to 3, and seven component scores are then summed to gain a global score [19]. A PSQI global score > 5 indicates poor sleep quality [19]. The traditional Chinese version of PSQI was used to assess sleep quality in this study [20]. The results of the PSQI questionnaire were retrospectively collected and analyzed. The CAT is a questionnaire for assessing the health status of COPD [17]. The test has eight items including cough, phlegm, chest tightness, breathlessness, activities, confidence, sleep, and energy. Each item can be scored as a single scale (from 0 to 5) with total scores ranging from 0 to 40. Higher CAT scores indicate poorer health. The modified Medical Research Council (mMRC) scale include five statements for rating dyspnea from 0 to 4, and higher mMRC grades indicate more dyspnea [18]. According to the GOLD guidelines, either a mMRC ≥ 2 or a CAT score ≥ 10 indicate more symptoms [15].

### Statistical analysis

Patient demographics, clinical characteristics, and medication use were summarized using descriptive statistics and data were expressed as mean ± standard deviation (SD). Comparisons between groups were made using the independent *t*-test, Chi-square test, one-way ANOVA, or Kruskal-Wallis one-way analysis of variance, where appropriate. Potential risk factors causing poor sleep quality in patients with COPD, including age, BMI, lung function, clinical symptoms, medications, medical history of comorbidities, and symptoms scores, were analyzed using univariate and multivariate analysis by applying a multiple logistic regression. All statistical analyses were performed using the using Statistical Analysis Software (SAS) version 9.3 (SAS Institute Inc., Cary, NC, USA).

## Results

A total of 377 stable patients with COPD and who attended the outpatient clinic and fulfilled the study criteria were analyzed. Table 1 shows the patients’ characteristics, including age, sex, BMI, FEV_1_% predicted, FVC% predicted, GOLD group, comorbidities, and current COPD medication. Most patients were elderly and 98 % were male. According to the GOLD classification, most patients were classified into GOLD groups B and D. Among those classified, 53 % had poor sleep quality (PSQI > 5).

**Table 1:** Demographic characteristics of the study population (*n* = 377)

Characteristics	Mean ± SD
Age, years	73.0 ± 10.1
Male sex, n (%)	371 (98.4 %)
BMI, (kg/m^2^)	23.6 ± 3.0
Smoking history	
Never smoker, n (%)	22 (6.0 %)
Ex-smoker, n (%)	232 (63.0 %)
Current smoker, n (%)	114 (31 %)
FEV_1_, L	1.4 ± 0.5
FEV_1_, predicted %	59.8 ± 23.0
Exacerbation in previous year, times	0.6 ± 1.2
Comorbidities	
Cardiovascular disease, n (%)	157 (41.6 %)
Ischemic heart disease, n (%)	35 (9.3 %)
Heart failure, n (%)	15 (4.0 %)
Hypertension, n (%)	129 (34.2 %)
Arrhythmia, n (%)	19 (5.0 %)
Depression, n (%)	10 (2.7 %)
Maintenance inhaler	
LABA, n (%)	18 (4.8 %)
LAMA, n (%)	87 (23.1 %)
LABA/LAMA, n (%)	21 (5.6 %)
LABA/ICS, n (%)	72 (19.1 %)
LAMA/ICS, n (%)	3 (0.8 %)
LABA/LAMA/ICS, n (%)	90 (23.9 %)
No maintenance inhaler, n (%)	86 (22.8 %)
GOLD classification	
Group A, n (%)	60 (15.9 %)
Group B, n (%)	150 (39.8 %)
Group C, n (%)	34 (9.0 %)
Group D, n (%)	133 (35.3 %)
PSQI score	
PSQI >5, n (%)	200 (53.1 %)
PSQI≦5, n (%)	177 (46.9 %)

*Abbreviations*: *BMI* body mass index, *GOLD* Global Initiative for Chronic Obstructive Lung Disease, *FEV*_*1*_ forced expiratory volume in the first second, *FVC* forced vital capacity, *ICS* inhaled corticosteroids, *LABA* long-acting beta agonists, *LAMA* long-acting muscarinic antagonist, *PSQI* Pittsburgh sleep quality index

Patients with COPD were divided by their pulmonary function tests according to the 2007 GOLD classification [16]. Based on the 2007 GOLD classification system, the percentages of subjects with PSQI scores greater than 5 among the four groups was not significant (Table 2).

**Table 2:** Measurements of COPD based on the 2007 GOLD classification

	GOLD Stage 1	GOLD Stage 2	GOLD Stage 3	GOLD Stage 4	*P*-value
	*N* = 68	*N* = 161	*N* = 123	*N* = 25	
MMRC, mean ± SD	1.50 ± 0.76	1.78 ± 0.75	1.96 ± 0.95	2.33 ± 1.09	<0.001
CAT, mean ± SD	9.15 ± 6.91	9.53 ± 6.35	11.07 ± 7.11	16.83 ± 8.29	<0.001
PSQI score, mean ± SD	6.54 ± 3.57	6.43 ± 3.50	6.33 ± 3.66	6.44 ± 3.16	0.970
PSQI >5, n (%)	36 (52.9)	85 (52.8)	64 (52.0)	15 (60.0)	0.910

*Abbreviations*: *CAT* COPD assessment test, *GOLD* Global Initiative for Chronic Obstructive Lung Disease, *mMRC* modified Medical Research Council, *PSQI*, Pittsburgh Sleep Quality Index

The COPD patients were also classified using the 2011 GOLD classification system [15], and the percentages of patients with PSQI >5 between the GOLD groups A, B, C, and D were 43.3, 54.0, 38.2, and 60.2 %, respectively (*p* < 0.05) (Table 3).

**Table 3:** Measurements of COPD based on 2011 GOLD classification

	GOLD group A	GOLD group B	GOLD group C	GOLD group D	*P*-value
	*N* = 60	*N* = 150	*N* = 34	*N* = 133	
MMRC, mean ± SD	0.87 ± 0.34	2.05 ± 0.58	0.72 ± 0.46	2.31 ± 0.81	<0.001
CAT, mean ± SD	4.52 ± 2.14	11.09 ± 6.75	4.74 ± 2.69	13.84 ± 7.08	<0.001
PSQI score, Mean ± SD	5.63 ± 2.92	6.67 ± 3.68	5.03 ± 3.29	6.85 ± 3.57	0.008
PSQI >5, n (%)	26 (43.3 %)	81 (54.0 %)	13 (38.2 %)	80 (60.2 %)	0.046

*Abbreviations*: *CAT* COPD assessment test, *GOLD* Global Initiative for Chronic Obstructive Lung Disease, *mMRC* modified Medical Research Council, *PSQI* Pittsburgh Sleep Quality Index

Table 4 demonstrated the specific components obtained from the PSQI. Among the seven components, sleep disturbance achieved the highest score compared with the other components. The most common sleep disturbances were “getting up to use the bathroom” (70.3 %), “wake up at night or early morning” (40.3 %) and “cough and snore loudly at night” (15.9 %). The percentage of the symptoms of sleep disturbance had no statistical difference between different stages of COPD according to the GOLD 2007 classification. (Table 5) We divided the subjects by the 2011 GOLD classification, patients had high percentage of “wake up at night or early morning” and “get up to use the bathroom” on GOLD group B and group D (Table 6).

**Table 4:** Sleep component scores based on the PSQI in patients with COPD

Components	Mean ± SD
Sleep quality	0.97 ± 0.75
Sleep latency	1.16 ± 1.09
Sleep duration	1.11 ± 0.94
Sleep efficiency	0.73 ± 1.04
Sleep disturbance	1.50 ± 0.59
Use of sleeping medication	0.47 ± 1.03
Daytime dysfunction	0.48 ± 0.66

*Abbreviation*: *PSQI* Pittsburgh Sleep Quality Index

**Table 5:** The distribution of sleep disturbances among patients with COPD based on 2007 GOLD classification

Items reporting ≥ 3 times per week	GOLD Stage 1	GOLD Stage2	GOLD Stage 3	GOLD Stage 4	*P*-value
	N (%)	N (%)	N (%)	N (%)	
Wake up at night or early morning	29 (42.6 %)	66 (41.0 %)	43 (35.0 %)	14 (56.0 %)	0.240
Get up to use the bathroom	45 (66.2 %)	118 (73.3 %)	80 (65.0 %)	22 (88.0 %)	0.085
Cannot breathe comfortably	2 (2.9 %)	11 (6.8 %)	11 (8.9 %)	4 (16.0 %)	0.161
Cough or snore loudly	10 (14.7 %)	23 (14.3 %)	21 (17.1 %)	6 (24.0 %)	0.628
Feel too cold	0 (0.0 %)	4 (2.5 %)	2 (1.6 %)	0 (0.0 %)	0.508
Feel too hot	0 (0.0 %)	4 (2.5 %)	2 (1.6 %)	1 (4.0 %)	0.512
Bad dreams	9 (13.2 %)	21 (13.0 %)	13 (10.6 %)	2 (8.0 %)	0.829
Pain	3 (4.4 %)	8 (5.0 %)	7 (5.7 %)	2 (8.0 %)	0.910

**Table 6:** The distribution of sleep disturbances among patients with COPD based on 2011 GOLD classification

Items reporting ≥ 3 times per week	GOLD group A	GOLD group B	GOLD group C	GOLD group D	*P*-value
	N (%)	N (%)	N (%)	N (%)	
Wake up at night or early morning	15 (25.0 %)	70 (46.7 %)	10 (29.4 %)	57 (42.9 %)	0.015
Get up to use the bathroom	37 (61.7 %)	114 (76.0 %)	19 (55.9 %)	95 (71.4 %)	0.047
Cannot breathe comfortably	0 (0.0 %)	11 (7.3 %)	2 (5.9 %)	15 (11.3 %)	0.050
Cough or snore loudly	8 (13.3 %)	21 (14.0 %)	4 (11.8 %)	27 (20.3 %)	0.383
Feel too cold	0 (0.0 %)	4 (2.7 %)	0 (0.0 %)	2 (1.5 %)	0.452
Feel too hot	0 (0.0 %)	4 (2.7 %)	0 (0.0 %)	3 (2.3 %)	0.487
Bad dreams	4 (6.7 %)	26 (17.3 %)	1 (2.9 %)	14 (10.5 %)	0.035
Pain	1 (1.7 %)	9 (6.0 %)	2 (5.9 %)	8 (6.0 %)	0.597

Table 7 shows the results of univariate analysis of variables associated with poor sleep quality (PSQI > 5) in patients with COPD based on logistic regression. Inhaled corticosteroids and the presence of a wheeze showed positive correlations with poor sleep quality scores with odds ratios (ORs) of 1.66 and 1.51, respectively. The CAT and MMRC scores also correlated with PSQI scores with ORs of 1.09 (95 % confidence interval [CI], 1.05–1.13) and 1.30 (95 % CI, 1.02–1.66), respectively. Upon multivariate analysis, the CAT score remained an independent factor in poor sleep quality with an OR of 1.09 (95 % CI, 1.04–1.14; *p* < 0.001).

**Table 7:** Variables associated with poor sleep quality among patients with COPD

Characteristics	Parameter estimate	Standard error	Odds ratio (95 % CI)	*P*-value
Univariate analysis				
Age	0.006	0.010	1.01 (0.99,1.03)	0.563
BMI	0.027	0.026	1.03 (0.98,1.08)	0.311
FEV_1_, predicted %	−0.003	0.004	1.00 (0.99,1.01)	0.555
Wheezing*	0.509	0.228	1.66 (1.06,2.60)	0.026
Theophylline	0.134	0.515	1.14 (0.42,3.14)	0.794
Oral steroids	−0.281	0.218	0.76 (0.49,1.16)	0.197
Inhaled LABA	−0.363	0.486	0.70 (0.27,1.80)	0.455
Inhaled LAMA	−0.369	0.245	0.69 (0.43,1.12)	0.133
Inhaled ICS*	0.412	0.210	1.51 (1.00,2.28)	0.049
Ischemic heart disease	0.312	0.362	1.37 (0.67,2.77)	0.389
Heart failure	−0.549	0.537	0.58 (0.20,1.66)	0.307
Hypertension	0.409	0.220	1.50 (0.98,2.32)	0.063
Arrhythmia	0.438	0.487	1.55 (0.60,4.03)	0.368
Depression	1.294	0.797	3.65 (0.76,17.4)	0.105
Anxiety	−0.124	0.823	0.88 (0.18,4.43)	0.880
CAT score*	0.085	0.017	1.09 (1.05,1.13)	<0.001
mMRC score*	0.264	0.125	1.30 (1.02,1.66)	0.035

*Abbreviations*: *BMI* body mass index, *FEV*_*1*_ forced expiratory volume in the first second, *ICS* inhaled corticosteroids, *LABA* long-acting beta agonist, *LAMA* long-acting muscarinic antagonist, *CAT* COPD assessment test, *mMRC* modified Medical Research Council, *CI* confidence interval

*p < 0.05

To explore the factors contributing to poor sleep quality using the CAT score, the differences in CAT items between good and poor sleepers were further analyzed. Among all participants, 131 patients had all detailed specific items on COPD assessment tests recorded. Not surprisingly, “sleep problem” was one factor in poor sleepers. However, in addition, higher phlegm production was a significant factor in poor sleepers (Table 8).

**Table 8:** Specific items on COPD Assessment Test between patients with COPD with poor sleep quality (PSQI > 5) and those without poor sleep quality

	With poor sleep quality (*N* = 86)	Without poor sleep quality (*N* = 45)	*P*-value
Cough	2.0 ± 1.7	1.6 ± 1,3	0.123
Phlegm*	2.4 ± 1.6	1.8 ± 1.5	0.030
Chest tightness	1.4 ± 1.4	1.1 ± 1.5	0.286
Breathlessness	2.4 ± 1.7	2.2 ± 1.9	0.558
Activities	1.0 ± 1.4	1.0 ± 1.6	0.805
Confidence	1.1 ± 1.5	1.1 ± 1.7	0.953
Sleep*	2.1 ± 1.9	0.7 ± 1.4	<0.001
Energy	1.8 ± 1.6	1.4 ± 1.7	0.176
CAT score*	14.4 ± 8.1	11.0 ± 7.9	0.022

*p < 0.05

## Discussion

Our study demonstrated that 53 % of patients with COPD in our cohort had poor sleep quality (PSQI score > 5). A high prevalence of poor sleep quality in patients with COPD has been reported in previous studies [1, 2, 8]. In a recent real-world study, 78 % of patients with COPD reported night time disturbances as measured by the Jenkins Sleep Questionnaire [21]. In a telephone study, Ohayon reported COPD subjects had higher insomnia symptoms than non-COPD subjects (48.1 versus 27.6 % respectively) [22].

Our study also confirmed sleep disturbances, as well as cough and dyspnea, as major concerns in patients with COPD. Some researchers suggest that poor sleep quality might result in cognitive dysfunction, depression, anxiety, poor survival and poor quality of life [7]. Determining which factors are associated with sleep disturbances in patients with COPD may improve their treatment strategy.

A high prevalence of sleep symptoms among patients with COPD was discovered approximately thirty years ago [23] but poor sleep quality remained a forgotten dimension of COPD until recently [24]. Few studies, however, have addressed factors such as medical history, pulmonary function tests, COPD medication, and questionnaires when analyzing sleep disturbances in COPD patients.

In this study, poor sleep quality was defined according to PSQI scores and included several symptoms. Interestingly, the most common symptoms that disturbed sleep in our COPD cohort were “getting up to use the bathroom” (69.9 %) and “wake up at night or early morning” (40.3 %) whereas “cough and snore loudly at night” or “bad dream” comprised the third and fourth complaints (15.9 and 11.9 %, respectively). Breathlessness, cough, sputum production, and wheezing were also among the most common symptoms in patients with COPD. In our study, we found the GOLD guideline updated in 2011, which was classified by their symptoms as well as lung function were related to sleep disturbance as shown in Tables 2, 3, 5, and 6. More symptoms patients (group B or group D; mMRC ≥ 2 or CAT ≥ 10) had a higher percentage of sleep disturbance including wake up at night or early morning, get up to use the bathroom, bad dream and cannot breathe comfortably.

In a nation-wide longitudinal population study, the overall prevalence of nocturnal voiding was 56 % at 50 years of age increasing to 74 % 10 years later [25]. The average age of our cohort was 73 years of age with 69.9 % complaining of nocturnal voiding symptoms, similar to the results from the population study. An epidemiological study of nocturia by Yoshimura et al. suggested that, besides benign prostate hypertrophy, nocturia correlated with age, race, medical problems (such as hypertension, diabetes, and stroke) psychological problems, quality of life and mortality [26]. Stephenson et al. reported that use of inhaled anticholinergic medications was associated with an increased risk of acute urinary retention in men with COPD, especially in those patients with benign prostate hyperplasia [27]. However, another study concluded that tiotropium did not affect lower urinary tract function in patients with COPD [28]. In our study, the use of inhaled long-acting muscarinic antagonists was not associated with poor sleep quality in the COPD patients. In patients with heart failure, nocturia is common and associated with sleep disturbance [29]. Heart failure may be one of the factors contributing to sleep disturbance in the patients with COPD. Although the most common cause of sleep disturbance was getting up to use the bathroom, patients with poor sleep quality also had more complaints of “wake up at night or early morning”, “cough or snore loudly”, and “cannot breathe comfortably” compared with those patients without poor sleep quality. Thus, the current study offers physicians significant information regarding ways to discover and solve sleep problems in patients with COPD. In a national epidemiology survey in Taiwan [30], males were predominated, especially in current and former smokers from 91 to 84 %. Our result had similar male predominant population which may due to our patients are mostly current or formal smokers.

Multivariate analysis showed that high CAT scores predicted poor sleep quality. These results implied that surveying the causes of sleep disturbance was important for patients with CAT scores > 10. Mehta et. al also reported that COPD patients with high CAT scores had a higher frequency of insomnia than those with low CAT scores [31]. A previous study reported an increased prevalence of night symptoms as the severity of airflow limitation increased [32]. In contrast, another study found that FEV_1_ was not associated with disturbed sleep nor with health related quality of life [7, 33]. In our study, we found no relationship between pulmonary function tests and poor sleep quality. This result implies that poor sleep quality is not directly related to pulmonary function tests. However, other underlying diseases, medical comorbidities, and adverse effects of drug therapy may be associated factors.

The causes of sleep disturbance in patients with COPD are multifactorial, includes respiratory symptoms [34], obstructive sleep apnea (OSA), psychiatric disorders, and medication-related insomnia [9, 35]. Sleep disturbances due to COPD medications is a common problem, and oral steroids, inhaled steroids, beta agonists, and theophylline have been implicated [10, 36–38]. In our studies, the presence of wheeze, inhaled ICS, and significant phlegm production contributed to poor sleep quality. After multivariate analysis, however, no single medication caused poor sleep quality; only the CAT score predicted poor sleep quality in our COPD population. “Much phlegm” on the CAT questionnaire in our cohort was significant in poor sleepers. The CAT score is a simple questionnaire developed to assess and monitor the symptoms of COPD [17]. We recommend the use of the CAT questionnaire rather than the MMRC to monitor symptoms of COPD because sleep disturbance is a major complaint in patients with COPD.

Our study had several limitations. We did not perform polysomnography to rule out sleep disorders such as OSA. Although OSA is prevalent in some selected COPD patients, the prevalence rate of OSA in patients with COPD was similar to the general population in the other reports [24, 39]. In addition, not all the factors which disturbed sleep (such as depression) were collected. The subjects included in our study may have had more medical comorbidities (and the percentage of GOLD B and D patients may have been higher) than those found in the general COPD population. Finally, the study was retrospective and the most data were collected in older men with COPD.

## Conclusions

More than half of our patients with COPD experienced poor sleep quality. The most common sleep complaints included getting up for the bathroom, waking up at night or in the early morning, and coughing or snoring loudly. Symptoms including wheezing, phlegm, and the use of inhaled corticosteroid may also have contributed to poor sleep quality in these COPD patients.

## Abbreviations

BMI, body mass index; CAT, COPD Assessment Test; COPD, chronic obstructive lung disease; FEV1, forced expiratory volume in the first second; FVC, forced vital capacity; GOLD, Global Initiatives for Chronic Obstructive Lung Disease; ICS, inhaled corticosteroids; LABA, long-acting beta agonists; LAMA, long-acting muscarinic antagonist; mMRC, Modified Medical Research Council; PSQI, Pittsburgh Sleep Quality Index; REM, rapid eye movement

## References

[1] Nunes DM, Mota RM, de Pontes Neto OL, Pereira ED, de Bruin VM, de Bruin PF (2009). Impaired sleep reduces quality of life in chronic obstructive pulmonary disease. Lung.

[2] Scharf SM, Maimon N, Simon-Tuval T, Bernhard-Scharf BJ, Reuveni H, Tarasiuk A (2011). Sleep quality predicts quality of life in chronic obstructive pulmonary disease. Int J Chron Obstruct Pulmon Dis.

[3] Valipour A, Lavie P, Lothaller H, Mikulic I, Burghuber OC (2011). Sleep profile and symptoms of sleep disorders in patients with stable mild to moderate chronic obstructive pulmonary disease. Sleep Med.

[4] Fleetham J, West P, Mezon B, Conway W, Roth T, Kryger M (1982). Sleep, arousals, and oxygen desaturation in chronic obstructive pulmonary disease. The effect of oxygen therapy. Am Rev Respir Dis.

[5] Mulloy E, McNicholas WT (1996). Ventilation and gas exchange during sleep and exercise in severe COPD. Chest.

[6] Ballard RD, Clover CW, Suh BY (1995). Influence of sleep on respiratory function in emphysema. Am J Respir Crit Care Med.

[7] Omachi TA, Blanc PD, Claman DM, Chen H, Yelin EH, Julian L, Katz PP (2012). Disturbed sleep among COPD patients is longitudinally associated with mortality and adverse COPD outcomes. Sleep Med.

[8] Lewis CA, Fergusson W, Eaton T, Zeng I, Kolbe J (2009). Isolated nocturnal desaturation in COPD: prevalence and impact on quality of life and sleep. Thorax.

[9] McNicholas WT, Calverley PM, Lee A, Edwards JC (2004). Long-acting inhaled anticholinergic therapy improves sleeping oxygen saturation in COPD. Eur Respir J.

[10] Roehrs T, Merlotti L, Halpin D, Rosenthal L, Roth T (1995). Effects of theophylline on nocturnal sleep and daytime sleepiness/alertness. Chest.

[11] Mastronarde JG, Wise RA, Shade DM, Olopade CO, Scharf SM (2008). Sleep quality in asthma: results of a large prospective clinical trial. J Asthma.

[12] Chrousos GA, Kattah JC, Beck RW, Cleary PA (1993). Side effects of glucocorticoid treatment. Experience of the Optic Neuritis Treatment Trial. JAMA.

[13] Calverley PM, Anderson JA, Celli B, Ferguson GT, Jenkins C, Jones PW, Yates JC, Vestbo J (2007). Salmeterol and fluticasone propionate and survival in chronic obstructive pulmonary disease. N Engl J Med.

[14] Fitzpatrick MF, Mackay T, Driver H, Douglas NJ (1990). Salmeterol in nocturnal asthma: a double blind, placebo controlled trial of a long acting inhaled beta 2 agonist. BMJ.

[15] Vestbo J, Hurd SS, Agusti AG, Jones PW, Vogelmeier C, Anzueto A, Barnes PJ, Fabbri LM, Martinez FJ, Nishimura M (2013). Global strategy for the diagnosis, management, and prevention of chronic obstructive pulmonary disease: GOLD executive summary. Am J Respir Crit Care Med.

[16] Rabe KF, Hurd S, Anzueto A, Barnes PJ, Buist SA, Calverley P, Fukuchi Y, Jenkins C, Rodriguez-Roisin R, van Weel C (2007). Global strategy for the diagnosis, management, and prevention of chronic obstructive pulmonary disease: GOLD executive summary. Am J Respir Crit Care Med.

[17] Jones PW, Harding G, Berry P, Wiklund I, Chen WH, Kline Leidy N (2009). Development and first validation of the COPD Assessment Test. Eur Respir J.

[18] Mahler DA, Wells CK (1988). Evaluation of clinical methods for rating dyspnea. Chest.

[19] Buysse DJ, Reynolds CF, Monk TH, Berman SR, Kupfer DJ (1989). The Pittsburgh Sleep Quality Index: a new instrument for psychiatric practice and research. Psychiatry Res.

[20] Tsai PS, Wang SY, Wang MY, Su CT, Yang TT, Huang CJ, Fang SC (2005). Psychometric evaluation of the Chinese version of the Pittsburgh Sleep Quality Index (CPSQI) in primary insomnia and control subjects. Qual Life Res.

[21] Price D, Small M, Milligan G, Higgins V, Gil EG, Estruch J (2013). Impact of night-time symptoms in COPD: a real-world study in five European countries. Int J Chron Obstruct Pulmon Dis.

[22] Ohayon MM (2014). Chronic obstructive pulmonary disease and its association with sleep and mental disorders in the general population. J Psychiatr Res.

[23] Klink M, Quan SF (1987). Prevalence of reported sleep disturbances in a general adult population and their relationship to obstructive airways diseases. Chest.

[24] McNicholas WT, Verbraecken J, Marin JM (2013). Sleep disorders in COPD: the forgotten dimension. Eur Respir Rev.

[25] Hakkinen JT, Hakama M, Shiri R, Auvinen A, Tammela TL, Koskimaki J (2006). Incidence of nocturia in 50 to 80-year-old Finnish men. J Urol.

[26] Yoshimura K (2012). Correlates for nocturia: a review of epidemiological studies. Int J Urol.

[27] Stephenson A, Seitz D, Bell CM, Gruneir A, Gershon AS, Austin PC, Fu L, Anderson GM, Rochon PA, Gill SS (2011). Inhaled anticholinergic drug therapy and the risk of acute urinary retention in chronic obstructive pulmonary disease: a population-based study. Arch Intern Med.

[28] Miyazaki H, Suda T, Otsuka A, Nagata M, Ozono S, Hashimoto D, Nakamura Y, Inui N, Nakamura H, Chida K (2008). Tiotropium does not affect lower urinary tract functions in COPD patients with benign prostatic hyperplasia. Pulm Pharmacol Ther.

[29] Redeker NS, Adams L, Berkowitz R, Blank L, Freudenberger R, Gilbert M, Walsleben J, Zucker MJ, Rapoport D (2012). Nocturia, sleep and daytime function in stable heart failure. J Card Fail.

[30] Cheng SL, Chan MC, Wang CC, Lin CH, Wang HC, Hsu JY, Hang LW, Chang CJ, Perng DW, Yu CJ (2015). COPD in Taiwan: a National Epidemiology Survey. Int J Chron Obstruct Pulmon Dis.

[31] Mehta JR, Ratnani IJ, Dave JD, Panchal BN, Patel AK, Vala AU (2014). Association of psychiatric co-morbidities and quality of life with severity of chronic obstructive pulmonary disease. East Asian Arch Psychiatry.

[32] Agusti A, Hedner J, Marin JM, Barbe F, Cazzola M, Rennard S (2011). Night-time symptoms: a forgotten dimension of COPD. Eur Respir Rev.

[33] Pickard AS, Yang Y, Lee TA (2011). Comparison of health-related quality of life measures in chronic obstructive pulmonary disease. Health Qual Life Outcomes.

[34] Klink ME, Dodge R, Quan SF (1994). The relation of sleep complaints to respiratory symptoms in a general population. Chest.

[35] Martin RJ, Bartelson BL, Smith P, Hudgel DW, Lewis D, Pohl G, Koker P, Souhrada JF (1999). Effect of ipratropium bromide treatment on oxygen saturation and sleep quality in COPD. Chest.

[36] Moser NJ, Phillips BA, Guthrie G, Barnett G (1996). Effects of dexamethasone on sleep. Pharmacol Toxicol.

[37] Kraft M, Wenzel SE, Bettinger CM, Martin RJ (1997). The effect of salmeterol on nocturnal symptoms, airway function, and inflammation in asthma. Chest.

[38] Barnes PJ (1995). Inhaled glucocorticoids for asthma. N Engl J Med.

[39] Sanders MH, Newman AB, Haggerty CL, Redline S, Lebowitz M, Samet J, O'Connor GT, Punjabi NM, Shahar E (2003). Sleep and sleep-disordered breathing in adults with predominantly mild obstructive airway disease. Am J Respir Crit Care Med.

